# Evaluation of Antibiotic Treatment And Antibiotic De-Escalation in a Intensive Care Unit With Selective Digestive Decontamination

**DOI:** 10.1186/2197-425X-3-S1-A2

**Published:** 2015-10-01

**Authors:** C Sánchez Ramirez, L Caipe Balcázar, S Hípola Escalada, MA Hernández Viera, M Cabrera Santana, N Sangil Monroy, A Bordes Benitez, P Saavedra Santana, S Ruiz Santana

**Affiliations:** University Hospital of Gran Canaria Dr Negrín, Intensive Care Unit, Las Palmas de Gran Canaria, Spain; University Hospital of Gran Canaria Dr Negrín, Pharmacy Department, Las Palmas de Gran Canaria, Spain; University Hospital of Gran Canaria Dr Negrín, Microbiology Department, Las Palmas de Gran Canaria, Spain; University of Las Palmas de Gran Canaria, Mathematics and Informatics Deparment, Las Palmas de Gran Canaria, Spain

## Objective

To evaluate the appropriate use of antibiotics and their de-escalation (DE) to treat nosocomial infections in an Intensive Care Unit (ICU) with Selective Digestive Decontamination (SDD).

## Method

In a polyvalent ICU of 30 beds from October 1, 2011 to September 30, 2014 nosocomial infections (pneumonia, urinary tract infections, catheter-related bacteremia (BRC) and of unknown origin and secondary nosocomial bacteremia) were prospectively collected. ENVIN-HELICS diagnostic criteria were applied. Etiology, inflammatory response to infection, antibiotic treatment (ATB T), and treatment modifications according to culture results, was analyzed. SDD was applied to all admitted patients requiring endotracheal intubation over 48 hours. For each of the groups categorical variables were summarized as frequencies and percentages and number in means and standard deviations (SD) or median with interquartile ranges (IQR). Percentages were compared, as appropriate, with the Fisher´s exact test.or X^2^ test and medians with the Wilcoxon test for independent samples. For those variables that were associated with DE in the univariate analysis were entered into a logistic multidimensional analysis. The model obtained was expressed by p-values and odd-ratios, which were estimated by confidence intervals at 95%. a hypothesis test was considered statistically significant when p-value was less than .05.

## Results

**Figure 1:**
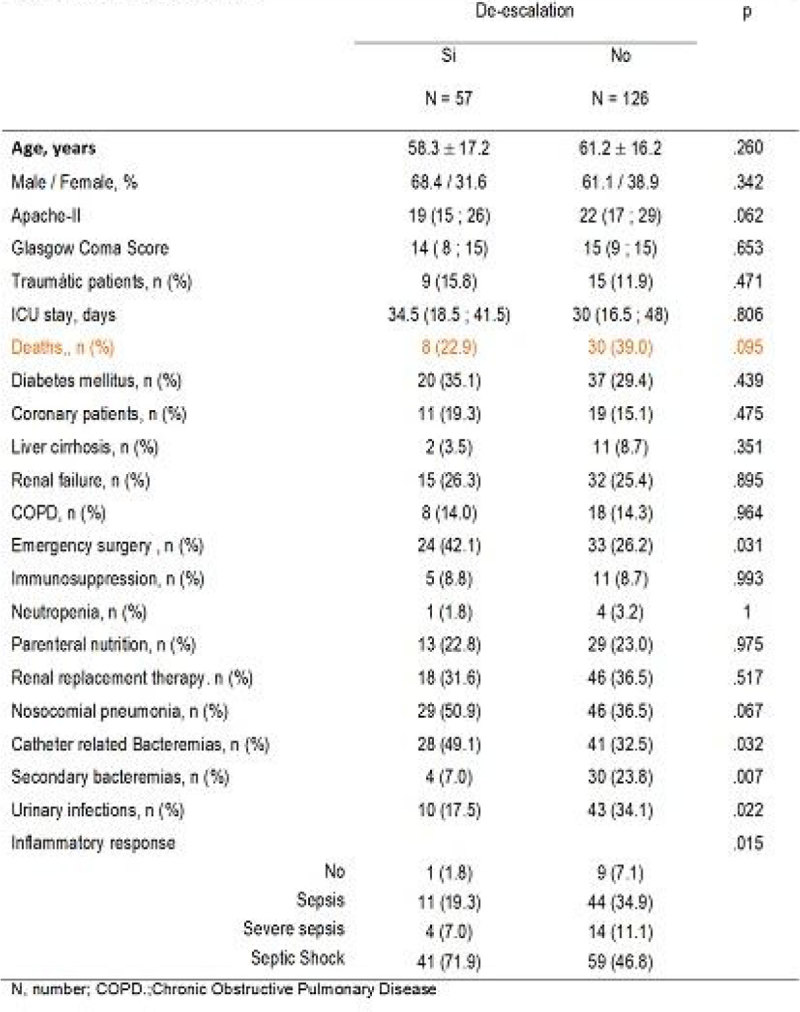
**Univariate analysis.**

Fifty-seven patients had ATB DE and 126 did not. There were no significant differences in demographics or type of admission (Figure 1)

**Figure 2:**
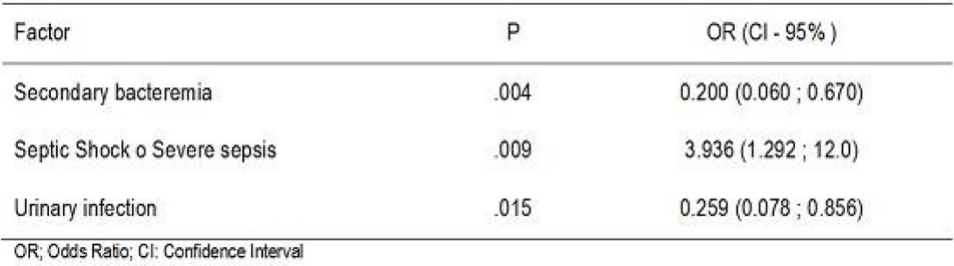
**Multivariate analysis.**

Mortality was lower in patients receiving DE antibiotic (ATB) (22.9%, p: 0.095). in the multivariate study of urinary tract infection, septic shock or severe sepsis and secondary bacteremia were significant (Figure 2).

**Figure 3:**
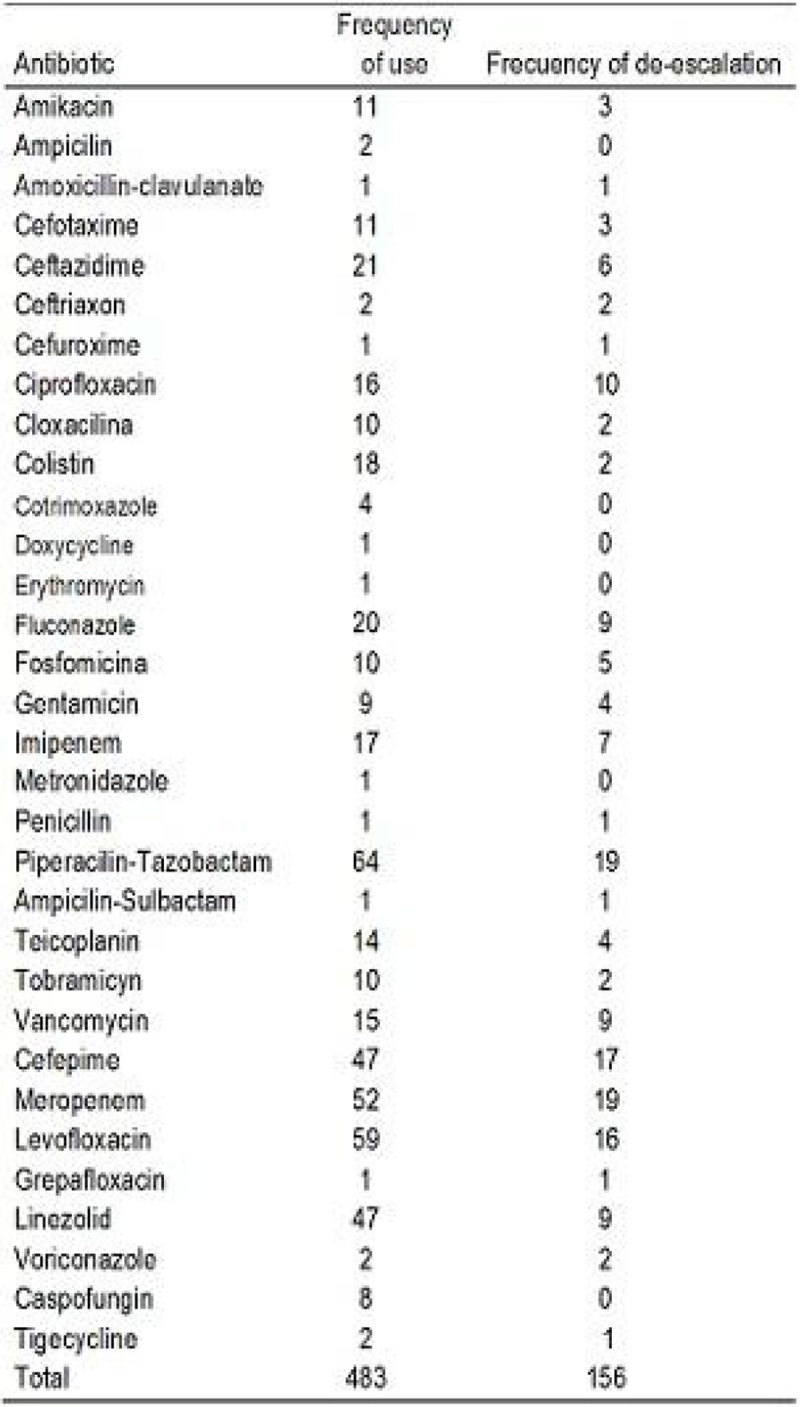
**Antibiotics.**

Of the 253 nosocomial infections ATB DE were performed at least in one of ATB in 131 (51.8%) of them. The inflammatory response and the type of infection did not show any decrease in the group with DE compared to all infections. The ATB T was inadequate in 43 infections (16.9%) (15 pneumonias, 11 urinary tract infections, 14 CRB and 3 secondary bacteremias). Targeted therapy was performed at least 1 time out of 109 infections (43.1% of infections), 28 pneumonias, 35 urinary tract infections, 38 CRB and 10 BRC secondary bacteremias. The number of antibiotics used was 483 and in 156 occasions ATB DE was performed. Frequency of use and DE is shown in Figure 3.

## Conclusions

Patients with received versus those that did not received DE had a tendency towards a significant lower mortality. The factors independently associated to DE were urinary and secondary bacteremia and septic shock or severe sepsis. Inadequate ATB T in our ICU happened in 16.9% of nosocomial infections. ATB DE was performed in 51.8% of them. The most commonly used antibiotics were piperacillin-tazobactam (29.6%), levofloxacin (12.2%) and meropenem (10.7%).

